# Integrating Philosophy of Understanding With the Cognitive Sciences

**DOI:** 10.3389/fnsys.2022.764708

**Published:** 2022-03-10

**Authors:** Kareem Khalifa, Farhan Islam, J. P. Gamboa, Daniel A. Wilkenfeld, Daniel Kostić

**Affiliations:** ^1^Department of Philosophy, Middlebury College, Middlebury, VT, United States; ^2^Independent Researcher, Madison, WI, United States; ^3^Department of History and Philosophy of Science, University of Pittsburgh, Pittsburgh, PA, United States; ^4^Department of Acute and Tertiary Care, University of Pittsburgh School of Nursing, Pittsburgh, PA, United States; ^5^Institute for Science in Society (ISiS), Radboud University, Nijmegen, Netherlands

**Keywords:** explanation, understanding, mechanism, computation, topology, dynamic systems, integration

## Abstract

We provide two programmatic frameworks for integrating philosophical research on understanding with complementary work in computer science, psychology, and neuroscience. First, philosophical theories of understanding have consequences about how agents should reason if they are to understand that can then be evaluated empirically by their concordance with findings in scientific studies of reasoning. Second, these studies use a multitude of explanations, and a philosophical theory of understanding is well suited to integrating these explanations in illuminating ways.

## Introduction

Historically, before a discipline is recognized as a science, it is a branch of philosophy. Physicists and chemists began their careers as “natural philosophers” during the Scientific Revolution. Biology and psychology underwent similar transformations throughout the nineteenth and early twentieth centuries. So, one might think philosophical discussions of understanding will be superseded by a “science of understanding.”

While we are no great forecasters of the future, we will suggest that philosophical accounts of understanding can make two important scientific contributions. First, they provide a useful repository of hypotheses that can be operationalized and tested by scientists. Second, philosophical accounts of understanding can provide templates for unifying a variety of scientific explanations.

We proceed as follows. We first present these two frameworks for integrating philosophical ideas about understanding with scientific research. Then we discuss the first of these frameworks, in which philosophical theories of understanding propose hypotheses that are tested and refined by the cognitive sciences. Finally, we discuss the second framework, in which considerations of understanding provide criteria for integrating different scientific explanations. Both of our proposals are intended to be programmatic. We hope that many of the relevant details will be developed in future work.

## Two Frameworks for Integration

**FIGURE 1:**
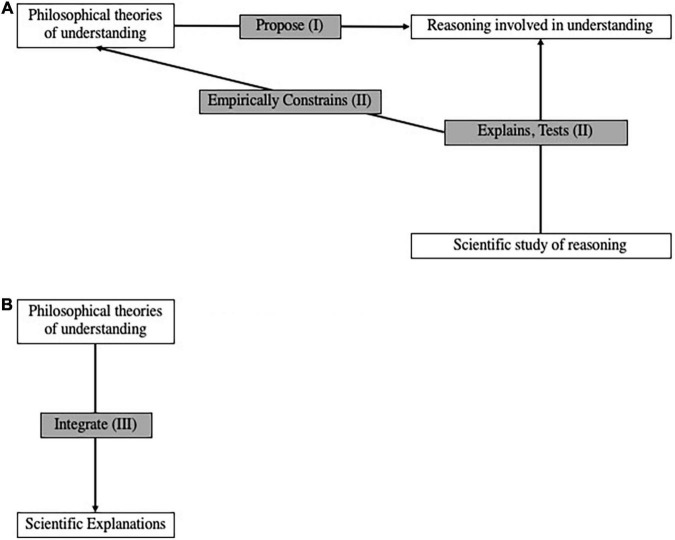
Two ways to integrate philosophical work on understanding with relevant sciences. **(A)** Naturalized epistemology of understanding. **(B)** Understanding-based integration.

As several reviews attest (Baumberger, 2014; Baumberger et al., 2016; Gordon, 2017; Grimm, 2021; Hannon, 2021), understanding has become a lively topic of philosophical research over the past two decades. While some work has been done to integrate these ideas with relevant findings from computer science, psychology, and neuroscience, these interdisciplinary pursuits are relatively nascent. While other frameworks are possible and should be developed, we propose two ways of effecting a more thoroughgoing synthesis between philosophy and these sciences (Figure 1). In the first framework for integrating philosophy with the cognitive sciences—what we call *naturalized epistemology of understanding* (Figure 1A)—the philosophy of understanding provides conjectures about reasoning that are tested and explained by the relevant sciences. In the second integrative framework—*understanding-based integration* (Figure 1B)—the philosophy of understanding provides broad methodological guidelines about how different kinds of scientific explanation complement each other. The two proposals are independent of each other: those unpersuaded by one may still pursue the other. We discuss each in turn.

## Naturalized Epistemology of Understanding

In epistemology, naturalism is the position that philosophical analyses of knowledge, justification, and kindred concepts should be intimately connected with empirical science. Different naturalists specify this connection in different ways; see Rysiew (2021) for a review. Given that philosophical interest in understanding has only recently achieved critical mass, the more specific research program of a naturalized epistemology of understanding is nascent. We propose to organize much existing work according to the framework in Figure 1A. More precisely, philosophical theories of understanding propose how reasoning operates in understanding (see section “Philosophical Theories Propose Reasoning in Understanding (I)”), and these proposals are constrained by explanations and empirical tests found in sciences that study this kind of reasoning (see section “Scientific Studies of Reasoning’s Contributions to the Philosophy of Understanding (II)”).

### Philosophical Theories Propose Reasoning in Understanding (I)

Two kinds of understanding have garnered significant philosophical attention: explanatory understanding (Grimm, 2010, 2014; Khalifa, 2012, 2013a,b,^2017^; Greco, 2013; Strevens, 2013; Hills, 2015; Kuorikoski and Ylikoski, 2015; Potochnik, 2017) and objectual understanding (Kvanvig, 2003; Elgin, 2004, 2017; Carter and Gordon, 2014; Kelp, 2015; Baumberger and Brun, 2017; Baumberger, 2019; Dellsén, 2020; Wilkenfeld, 2021). Explanatory understanding involves understanding why or how something is the case. (For terminological convenience, subsequent references to “understanding-why” are elliptical for “understanding-why or –how.”) Examples include understanding why Caesar crossed the Rubicon and understanding how babies are made. Objectual understanding is most easily recognized by its grammar: it is the word “understanding” followed immediately by a noun phrase, e.g., understanding Roman history or understanding human reproduction. Depending on the author, the objects of objectual understanding are taken to be subject matters, phenomena, and for some authors (e.g., Wilkenfeld, 2013), physical objects and human behaviors. For instance, it is natural to think of Roman history as a subject matter but somewhat counterintuitive to think of it as a phenomenon. It is more natural to think of, e.g., the unemployment rate in February 2021 as a phenomenon than as a subject matter. Human reproduction, by contrast, can be comfortably glossed as either a subject matter or a phenomenon.

To clarify what they mean by explanatory and objectual understanding, philosophers have disambiguated many other senses of the English word “understanding.” Frequently, these senses are briefly mentioned to avoid confusion but are not discussed at length. They are listed in Table 1. Scientists may find these distinctions useful when characterizing the kind of understanding they are studying. That said, we will focus on explanatory understanding hereafter. Thus, unless otherwise noted, all subsequent uses of “understanding” refer exclusively to explanatory understanding.

**TABLE 1:** Kinds of understanding that philosophers infrequently discuss (Khalifa, 2017, p. 2).

Kind of understanding	Typical complement	Examples
Propositional	That + declarative sentence	I understand that you might not enjoy reading this book.
Broad linguistic	Name of a language	Schatzi understands German.
Narrow linguistic	What + a linguistic expression + means	Schatzi understands what “Ich bin ein Berliner” means.
Procedural	How + infinitive	Miles understands how to play trumpet.
Non-explanatory interrogative	Embedded question that does not seek an explanation as its answer (most who, where, what, and when questions)	I understand who my friends are. I understand where my friends will be going. I understand what my friends are doing. I understand when my friends need a good laugh.

Virtually all philosophers agree that one can possess an accurate explanation without understanding it, e.g., through rote memorization. In cases such as this, philosophers widely agree that the lack of understanding is due to the absence of significant *inferential* or *reasoning* abilities. However, philosophers disagree about *which* inferences characterize understanding. Three broad kinds of reasoning have emerged. First, some focus on the reasoning required to *construct* or *consider* explanatory models (Newman, 2012, 2013, 2015; De Regt, 2017). Second, others focus on the reasoning required to *evaluate* those explanatory models (Khalifa, 2017). On both these views, explanatory models serve as the *conclusions* of the relevant inferences. However, the third and most prominent kind of reasoning discussed takes explanatory information as *premises* of the relevant reasoning—paradigmatically the inferences about how counterfactual changes in the explanatory variable or *explanans* would result in changes to the dependent variable or *explanandum* (Hitchcock and Woodward, 2003; Woodward, 2003; Grimm, 2010, 2014; Bokulich, 2011; Wilkenfeld, 2013; Hills, 2015; Kuorikoski and Ylikoski, 2015; Rice, 2015; Le Bihan, 2016; Potochnik, 2017; Verreault-Julien, 2017). This is frequently referred to as the ability to answer “what-if-things-had-been-different questions.” Many of these authors discuss all three of these kinds of reasoning—which we call *explanatory consideration, explanatory evaluation*, and *counterfactual reasoning*—often without explicitly distinguishing them in the ways we have here.

### Scientific Studies of Reasoning’s Contributions to the Philosophy of Understanding (II)

A naturalized epistemology of understanding begins with the recognition that philosophers do not have a monopoly on studying these kinds of reasoning. Computer scientists, psychologists, and neuroscientists take explanatory and counterfactual reasoning to be important topics of research. Undoubtedly, each discipline has important insights and contributions. Moreover, these scientific disciplines may raise interesting questions about understanding that are not on the current philosophical agenda.

Cognitive psychological investigations into the nature of explanation and understanding frequently focus on the role of those states in our cognitive lives. To the extent that one can derive a general lesson from this literature, it is probably that both having and seeking explanations aid other crucial cognitive tasks such as prediction, control, and categorization. Developmental psychologists argue that having proper explanations promotes survival, and that at least the sense of understanding evolved to give us an immediate reward for gaining such abilities (Gopnik, 1998). In cognitive psychology, Koslowski et al. (2008) have argued that having an explanation better enables thinkers to incorporate evidence into a causal framework. Lombrozo and collaborators have done extensive empirical work investigating the epistemic advantages and occasional disadvantages of simply being prompted to explain new data. They find that under most normal circumstances trying to seek explanations enables finding richer and more useful patterns (Williams and Lombrozo, 2010). This work also has the interesting implication that the value of explanation and understanding depends on the extent to which there are genuine patterns in the world, with fully patterned worlds granting the most advantages from prompts to explain (ibid.), and more exception-laden worlds providing differential benefits (Kon and Lombrozo, 2019). It has also been demonstrated that attempts to explain can (perhaps counterintuitively) systematically mislead. For example, attempts to explain can lead to miscategorization and inaccurate predictions when there are no real patterns in the data (Williams et al., 2013). Similarly, laypeople can be misguided by the appearance of irrelevant neuroscientific or otherwise reductive explanations (Weisberg et al., 2008; Hopkins et al., 2016). In more theoretical work, Lombrozo (2006) and Lombrozo and Wilkenfeld (2019) consider how different kinds of explanation can lead to understanding that is either more or less tied to specific causal pathways connecting explananda and explanantia vs. understanding focused on how different pathways can lead to the same end result. Thagard (2012) has argued that explanatory reasoning is key to science’s goals both intrinsically and as they contribute to truth and education.

One recent thread in the cognitive science and philosophy of understanding combines insights from information theory and computer science to characterize understanding in terms of data compression. Data compression (Grünwald, 2004) involves the ability to produce large amounts of information from relatively shorter hypotheses and explicitly encoded data sets---in computer science and model-centric physics, there is a burgeoning sense that understanding is tied to pattern recognition and data compression. Petersen (2022)^1^ helpfully documents an array of such instances. Li and Vitányi (2008) use compression and explanation almost interchangeably, and at some points even suggest a possible equivalence between compression and the scientific endeavor generally, as in Davies (1990). Tegmark (2014) likewise connects the notion of compression with the explanatory goals of science. Wilkenfeld (2019) translates the importance of compression to good scientific (and non-scientific) understanding into the idiom of contemporary philosophy of science. While part of the inspiration characterizing understanding in terms of compression comes from the traditional “unificationist” philosophical position that understanding involves having to know fewer brute facts (Friedman, 1974) or argument patterns (Kitcher, 1989), the introduction of compression helps evade some objections to unificationist views, such as the fact that such views require explanations to be arguments (Woodward, 2003) and the fact that they allow for understanding *via* unification that no actual human agent can readily use (Humphreys, 1993). [Compression as a marker for intelligence has come under recent criticism (e.g., Chollet, 2019) as only accounting for past data and not future uncertainties; we believe Wilkenfeld’s (2019) account evades this criticism by defining the relevant compression partially in terms of usefulness, but defending that claim is beyond the scope of this paper.]

There has also been more direct work on leveraging insights from computer science in order to try to build explanatory schemas and even to utilize those tools to reach conclusions about true explanations. Schank (1986) built a model of computerized explanations in terms of scripts and designed programs to look for the best explanations. Similarly, Thagard (1989, 1992, 2012)—who had previously (Thagard, 1978) done seminal philosophical work on good-making features of explanation and how they should guide theory choice—attempted to automate how computers could use considerations of explanatory coherence to make inferences about what actually occurred.

One underexplored area in the philosophy of understanding and computer science is the extent to which neural nets and deep learning machines can be taken to understand anything. While Turing (1950) famously argued that a machine that could behave sufficiently close to a person could thereby think (and thus, perhaps, understand), many argue that learning algorithms are concerned with prediction *as opposed to* understanding. The most extreme version of this position is Searle’s (1980) claim that computers by their nature cannot achieve understanding because it requires semantic capacities when manipulating symbols (i.e., an ability to interpret symbols and operations, and to make further inferences based on those interpretations). Computers at best have merely syntactic capabilities (they can manipulate symbols using sets of instructions, without understanding the meaning of either symbols or operation upon them). However, at the point where deep learning machines have hidden representations (Korb, 2004), can generate new (seemingly theoretical) variables (ibid.), and can be trained to do virtually any task to which computer scientists have set their collective minds (including what looks from the outside like abstract reasoning in IBM’s Watson and their Project Debater), it raises vital philosophical questions regarding on what basis we can continue to deny deep learning machines the appellation of “understander.”

Elsewhere in cognitive science, early psychological studies of reasoning throughout the 1960s and 1970s focused on deductive reasoning and hypothesis testing (Osman, 2014). A major influence on this trajectory was Piaget’s (1952) theory of development, according to which children develop the capacity for hypothetico-deductive reasoning around age 12. The kinds of reasoning studied by psychologists then expanded beyond their logical roots to include more humanistic categories such as moral reasoning (Kohlberg, 1958). The psychology literature offers a rich body of evidence demonstrating how people reason under various conditions. For example, there is ample evidence that performance on reasoning tasks is sensitive to the semantic content of the problem being solved. One interpretation of this phenomenon is that in some contexts, people do not reason by applying content-free inference rules (Cheng and Holyoak, 1985; Cheng et al., 1986; Holyoak and Cheng, 1995). This empirical possibility is of particular interest for philosophers. In virtue of their (sometimes extensive) training in formal logic, philosophers’ reasoning practices may be atypical of the broader population. This in turn may bias their intuitions about how “people” or “we” reason in various situations, including when understanding. Another issue raised by sensitivity to semantic content is how reasoning shifts depending on the object of understanding. Although the distinctions explicated by philosophers (e.g., explanatory vs. objectual understanding) are clear enough, it is an open empirical question whether and how reasoning differs *within* these categories depending on the particular object and other contextual factors. As a final example, a further insight from psychology is that people may have multiple modes of reasoning that can be applied to the very same problem. Since Wason and Evans (1974) suggested the idea, dual-process theories have dominated the psychology of reasoning.^2^ Although both terminology and precise hypotheses vary significantly among dual-process theories (Evans, 2011, 2012), the basic idea is that one system of reasoning is fast and intuitive, relying on prior knowledge, while another is slow and more cognitively demanding. Supposing two or more systems of reasoning can be deployed in the same situation, one important consideration is how they figure in theories about the reasoning involved in understanding. To the extent that philosophical accounts are not merely normative but also aim at describing how people actually reason when understanding, psychological studies provide valuable empirical constraints and theoretical considerations.

With the aid of techniques for imaging brains while subjects perform cognitive tasks, neuroscientists have also made great progress in recent decades on identifying regions of the brain involved in reasoning. While that is certainly a worthwhile goal, it may seem tangential to determining the kind of reasoning that characterizes understanding. Here, we suggest two ways in which findings from neuroscience may help with this endeavor. First, neuroscientific evidence can help resolve debates where behavioral data underdetermine which psychological theory is most plausible. More precisely, in cases where competing psychological models of reasoning make the same behavioral predictions, they can be further distinguished by the kinds of neural networks that would implement the processes they hypothesize (Operskalski and Barbey, 2017). For example, Goel et al. (2000) designed a functional magnetic resonance imaging (fMRI) experiment to test the predictions of dual mechanism theory vs. mental model theory. According to the former, people have distinct mechanisms for form- and content-based reasoning, and the latter should recruit language processing structures in the left hemisphere. Mental model theory, by contrast, claims that reasoning essentially involves iconic representations, i.e., non-linguistic representations whose structure corresponds to the structure of whatever they represent (Johnson-Laird, 2010). In early formulations of the theory, it was assumed that different kinds of reasoning problems depend on the same visuo-spatial mechanisms in the right hemisphere (Johnson-Laird, 1995). Goel et al. (2000) tested the theories against one another by giving subjects logically equivalent syllogisms with and without semantic content. As expected, behavioral performance was similar in both conditions. Neither theory predicts significant behavioral differences. Consistent with both theories, the content-free syllogisms engaged spatial processing regions in the right hemisphere. However, syllogisms with semantic content activated a left hemisphere ventral network that includes language processing structures like Broca’s area. Unsurprisingly, proponents of mental models have disputed the interpretation of the data (Kroger et al., 2008). We do not take a stance on the issue here. We simply raise the case because it illustrates how neuroscience can contribute to debates between theories of reasoning pitched at the psychological level.

Neuroscientific evidence can also guide the revision of psychological models of understanding and reasoning. The broader point is about cognitive ontology. In the sense we mean here, a cognitive ontology is a set of standardized terms which refer to the entities postulated by a cognitive theory (Janssen et al., 2017). The point of developing a cognitive ontology is to represent the structure of psychological processes and facilitate communication through a shared taxonomy. One role for neuroscience is to inform the construction of cognitive ontologies. Price and Friston (2005), for instance, defend a strong bottom-up approach. In their view, components in a cognitive model (e.g., a model of counterfactual reasoning) should be included or eliminated depending on our knowledge of functional neuroanatomy. Others agree that neuroscience has a crucial role to play in theorizing about cognitive architecture but reject that it has any special authority in this undertaking (Poldrack and Yarkoni, 2016; Sullivan, 2017). We take no position here on how exactly neuroscience should influence the construction of cognitive models and ontologies. Instead, we highlight this important interdisciplinary issue to motivate the potential value of neuroscience for models of understanding and the reasoning involved in it, including those developed by philosophers.

## Philosophical Theories of Understanding Integrate Scientific Explanations (III)

Thus, there appear to be ample resources for a naturalized epistemology of understanding, in which explanations and empirical tests from the cognitive sciences empirically constrain philosophical proposals about the kinds of reasoning involved in understanding. However, we offer a second and distinct proposal for how the philosophy of understanding can inform scientific practice: as an account of how different explanations can be integrated (Figure 1B).

Such integration is needed when different explanations of a single phenomenon use markedly different vocabularies and concepts. This diversity of explanations is prevalent in several sciences—including the cognitive sciences. To that end, we first present different kinds of explanations frequently found in the cognitive sciences. Whether these different explanations are complements or competitors to each other raises several issues that are simultaneously methodological and philosophical. To address these issues, we then present a novel account of explanatory integration predicated on the idea that explanations are integrated to the extent that they collectively promote understanding. To illustrate the uniqueness of this account, we contrast our account of integration with a prominent alternative in the philosophical literature.

Before proceeding, two caveats are in order. First, although we focus on the cognitive sciences, the account of explanatory integration proposed here is perfectly general. In principle, the same account could be used in domains ranging from particle physics to cultural anthropology. Second, our aim is simply to show that our account of integration enjoys some initial plausibility; a more thoroughgoing defense exceeds the current paper’s scope.

### A Variety of Scientific Explanations

Puzzles about explanatory integration arise only if there are explanations in need of integration, i.e., explanations whose fit with each other is not immediately obvious. In this section, we provide examples of four kinds of explanations found in the cognitive sciences: mechanistic, computational, topological, and dynamical.

#### Mechanistic Explanations

Mechanistic explanations are widespread in the cognitive sciences (Bechtel and Richardson, 1993; Machamer et al., 2000; Craver, 2007; Illari and Williamson, 2010; Glennan, 2017; Craver and Tabery, 2019). Despite extensive discussion in the philosophical literature, there is no consensus on the proper characterization of mechanisms or how exactly they figure in mechanistic explanations.^3^ For our purposes, we illustrate basic features of mechanistic explanations by focusing on Glennan’s (2017, p. 17) minimal conception of mechanisms:

A mechanism for a phenomenon consists of entities (or parts) whose activities and interactions are organized so as to be responsible for the phenomenon.

This intentionally broad proposal captures a widely held consensus among philosophers about conditions that are necessary for something to be a mechanism. Where they disagree is about further details, such as the nature and role of causation, regularities, and levels of analysis involved in mechanisms. At a minimum, mechanistic explanations account for the phenomenon to be explained (the *explanandum*) by identifying the organized entities, activities, and interactions responsible for it.

Consider the case of the action potential. A mechanistic explanation of this phenomenon specifies parts such as voltage-gated sodium and potassium channels. It describes how activities of the parts, like influx and efflux of ions through the channels, underlie the rapid changes in membrane potential. It shows how these activities are organized such that they are responsible for the characteristic phases of action potentials. For example, the fact that depolarization precedes hyperpolarization is explained in part by the fact that sodium channels open faster than potassium channels. In short, mechanistic explanations spell out the relevant physical details.

Importantly, not all theoretical achievements in neuroscience are mechanistic explanations. As a point of contrast, compare Hodgkin and Huxley’s (1952) groundbreaking model of the action potential. With their mathematical model worked out, they were able to predict properties of action potentials and neatly summarize empirical data from their voltage clamp experiments. However, as Hodgkin and Huxley (1952) explicitly pointed out, their equations lacked a physical basis. There is some disagreement among philosophers about how we should interpret the explanatory merits of the model (Levy, 2014; Craver and Kaplan, 2020; Favela, 2020a), but what is clear is that the Hodgkin and Huxley model is a major achievement that is *not* a mechanistic explanation of the action potential. We return to issues such as these below.

#### Computational Explanations

Mechanistic explanations are sometimes contrasted with other kinds of explanation. In the philosophical literature, computational explanations are perhaps the most prominent alternative. Computational explanations are frequently considered a subset of *functional explanations*. The latter explain phenomena by appealing to their function and the functional organization of their parts (Fodor, 1968; Cummins, 1975, 1983, 2000). Insofar as computational explanations are distinct from other kinds of functional explanations, it is because the functions to which they appeal involve information processing. Hereafter, we focus on computational explanations.

In computational explanations, a phenomenon is explained in terms of a system performing a computation. A computation involves the processing of input information according to a series of specified operations that results in output information. While many computational explanations describe the object of computation as having representational content, some challenge this as a universal constraint on computational explanations (Piccinini, 2015; Dewhurst, 2018; Fresco and Miłkowski, 2021). We will use “information” broadly, such that we remain silent on this issue. Here, “operations” refer to logical or mathematical manipulations on information such as addition, subtraction, equation (setting a value equal to something), “AND,” etc. For example, calculating *n!* involves taking in input *n* and calculating the product of all natural numbers less than or equal to *n* and then outputting said product. Thus, we can explain why pressing “5,” “!,” “=”, in sequence on a calculator results in the display reading “120”; the calculator *computes* the factorial.

More detailed computational explanations of this procedure are possible. For example, the calculator performs this computation by storing *n* and iteratively multiplying the stored variable by one less than the previous iteration from *n* to 1. In this case, the operations being used are equation, multiplication, and subtraction. The information upon which those operations are being performed are the inputted value for *n* and the stored variable for the value of the factorial at that iteration.

#### Topological Explanations

In topological or “network” explanations, a phenomenon is explained by appeal to graph-theoretic properties. Scientists infer a network’s structure from data, and then apply various graph-theoretic algorithms to measure its topological properties. For instance, clustering coefficients measure degrees of interconnectedness among nodes in the same neighborhood. Here, a node’s *neighborhood* is defined as the set of nodes to which it is directly connected. An individual node’s *local* clustering coefficient is the proportion of edges within its neighborhood divided by the number of edges that could possibly exist between the members of its neighborhood. By contrast, a network’s *global* clustering coefficient is the ratio of closed triplets to the total number of triplets in a graph. A triplet of nodes is any three nodes that are connected by at least two edges. An *open* triplet is connected by exactly two edges; a *closed* triplet, by three. Another topological property, average (or “characteristic”) path length, measures the mean number of edges needed to connect any two nodes in the network.

In their seminal paper, Watts and Strogatz (1998) applied these concepts to a family of graphs and showed how a network’s topological structure determines its dynamics. First, *regular graphs* have both high global clustering coefficients and high average path length. By contrast, *random graphs* have low global clustering coefficients and low average path length. Finally, they introduced a third type of *small-world graph* with high clustering coefficient but low average path length.

Highlighting differences between these three types of graphs yields a powerful explanatory strategy. For example, because regular networks have larger average path lengths than small-world networks, things will “diffuse” throughout the former more slowly than the latter, largely due to the greater number of edges to be traversed. Similarly, because random networks have smaller clustering coefficients than small-world networks, things will also spread throughout the former more slowly than the latter, largely due to sparse interconnections within neighborhoods of nodes. Hence, *ceteris paribus*, propagation/diffusion is faster in small-world networks. This is because the fewer long-range connections between highly interconnected neighborhoods of nodes shorten the distance between neighborhoods of nodes that are otherwise very distant and enables them to behave as if they were first neighbors. For example, Watts and Strogatz showed that the nervous system of *Caenorhabditi elegans* is a small-world network, and subsequent researchers argued that this system’s small-world topology explains its relatively efficient information propagation (Latora and Marchiori, 2001; Bullmore and Sporns, 2012).

#### Dynamical Explanations

In dynamical explanations, phenomena are accounted for using the resources of dynamic systems theory. At root, a system is dynamical if its state space can be described using differential equations, paradigmatically of the following form:


$$\dot{\text{x}}\,\left(t\right)=\text{f}\,\left(\text{x}\,\left(t\right);p,t\right)$$

Here, *x* is a vector (often describing the position of the system of interest), ***f*** is a function, *t* is time, and ***p*** is a fixed parameter. Thus, the equation describes the evolution of a system over time. In dynamical explanations, these equations are used to show how values of a quantity at a given time and place would uniquely determine the phenomenon of interest, which is typically treated as values of the same quantity at a subsequent time.

For example, consider dynamical explanations of why bimanual coordination—defined roughly as wagging the index fingers of both hands at the same time—is done either in- or anti-phase. Haken et al. (1985) use the following differential equation to model this phenomenon:


$$\frac{d\,\phi }{d\,t}=-a\,s\,i\,n\,\phi -2\,b\,s\,i\,n\,2\,\phi$$

Here ϕ is relative phase, having a value of either 0° or 180° (representing in- and anti-phase conditions, respectively) and *b/a* is the coupling ratio inversely related to the oscillations’ frequency. The explanation rests on the fact that only the in- and anti-phase oscillations of the index fingers are basins of attraction.

### Understanding-Based Integration

Thus far, we have surveyed four different kinds of explanation—mechanistic, computational, topological, and dynamical. Moreover, each seems to have some explanatory power for some phenomena. This raises the question as to how these seemingly disparate kinds of explanation can be integrated. We propose a new account of “understanding-based integration” (UBI) to answer this question. A clear account of understanding is needed if it is to integrate explanations. To that end, we first present Khalifa’s (2017) model of understanding. We then extend this account of understanding to provide a framework for explanatory integration.

#### An Account of Understanding

We highlight two reasons to think that Khalifa’s account of understanding is especially promising as a basis for explanatory integration. First, as Khalifa (2019) argues, his is among the most demanding philosophical accounts of understanding. Consequently, it serves as a useful ideal to which scientists should aspire. Second, this ideal is not utopian. This is especially clear with Khalifa’s requirement that scientists evaluate their explanations relative to the best available methods and evidence. Indeed, among philosophical accounts of understanding, Khalifa’s account is uniquely sensitive to the centrality of hypothesis testing and experimental design in advancing scientific understanding (Khalifa, 2017; Khalifa, in press), and thus makes contact with workaday scientific practices. In this section, we present its three core principles.

Khalifa’s first central principle is the *Explanatory Floor*:

Understanding why *Y* requires possession of a correct explanation of why *Y*.

The Explanatory Floor’s underlying intuition is simple. It seems odd to understand why *Y* while lacking a correct answer to the question, “Why *Y*?” For instance, the person who lacks a correct answer to the question “Why do apples fall from trees?” does not understand why apples fall from trees. Since explanations are answers to why-questions, the Explanatory Floor appears platitudinous. Below, we provide further details about correct explanation.

The Explanatory Floor is only one of three principles comprising Khalifa’s account and imposes only a necessary condition on understanding. By contrast, the second principle, the *Nexus Principle*, describes how understanding can improve:

Understanding why *Y* improves in proportion to the amount of correct explanatory information about *Y* (= *Y’s* explanatory nexus) in one’s possession.

To motivate the Nexus Principle, suppose that one person can correctly identify two causes of a fire, and another person can only identify one of those causes. *Ceteris paribus*, the former understands why the fire occurred better than the latter. Crucially in what follows, however, “correct explanatory information” is not limited to correct explanations. The explanatory nexus also includes the *relationships* between correct explanations. We return to these “inter-explanatory relationships” below.

Furthermore, recall our earlier remark that gaps in understanding arise when one simply has an accurate representation of an explanation (or explanatory nexus) without significant cognitive ability. This leads to the last principle, the *Scientific Knowledge Principle*:

Understanding why *Y* improves as one’s possession of explanatory information about *Y* bears greater resemblance to scientific knowledge of *Y*’s explanatory nexus.

Once again, we may motivate this with a simple example. Consider two agents who possess the same explanatory information that nevertheless differ in understanding because of their abilities to relate that information to relevant theories, models, methods, and observations. The Scientific Knowledge Principle is intended to capture this idea. Khalifa provides a detailed account of scientific knowledge of an explanation:

An agent *S* has scientific knowledge of why *Y* if and only if there is some *X* such that *S*’s belief that *X explains Y* is the safe result of *S*’s scientific explanatory evaluation (SEEing).

The core notions here are safety and SEEing. Safety is an epistemological concept that requires an agent’s belief to not easily have been false given the way in which it was formed (Pritchard, 2009). SEEing then describes the way a belief in an explanation should be formed to promote understanding. SEEing consists of three phases:

1. *Considering* plausible potential explanations of how/why *Y*;
2. *Comparing* those explanations using the best available methods and evidence; and
3. Undertaking *commitments* to these explanations on the basis these comparisons. Paradigmatically, commitment entails that one believes only those plausible potential explanations that are decisive “winners” at the phase of comparison.

Thus, scientific knowledge of an explanation is achieved when one’s commitment to an explanation could not easily have been false given the way that one considered and compared that explanation to plausible alternative explanations of the same phenomenon.

#### Understanding-Based Integration

With our account of understanding in hand, we now argue that it provides a fruitful account of how different explanations, such as the ones discussed above, can be integrated. The Nexus Principle is the key engine of integration. As noted above, this principle states that understanding improves in proportion to the amount of explanatory information possessed. In the cognitive sciences, a multitude of factors explain a single phenomenon. According to the Nexus Principle, understanding improves not only when more of these factors are identified, but when the “inter-explanatory relationships” between these factors are also identified.

One “inter-explanatory relationship” is that of *relative goodness*. Some explanations are *better* than others, even if both are correct. For instance, the presence of oxygen is explanatorily relevant to any fire’s occurrence. However, oxygen is rarely judged as the *best* explanation of a fire. Per the Nexus Principle, grasping facts such as these enhances one’s understanding. Parallel points apply in the cognitive sciences. For example, it has been observed that mental simulations that involve episodic memory engage the default network significantly more than mental simulations that involve semantic memory (Parikh et al., 2018). Hence, episodic memory better explains cases in which the default network was more active during a mental simulation than does semantic memory.

**FIGURE 2:**
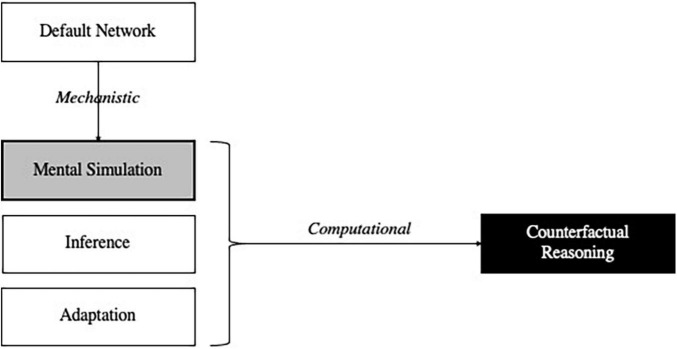
Computational and mechanistic explanations involved in counterfactual reasoning. Mental simulation (gray box) both contributes to the computational explanation of counterfactual reasoning (black box) and is mechanistically explained by the activation of the default network.

However, correct explanations can stand in other relations than superiority and inferiority. For instance, the aforementioned explanation involving the default network contributes to a more encompassing computational explanation of counterfactual reasoning involving three core stages of counterfactual thought (Van Hoeck et al., 2015). First, alternative possibilities to the actual course of events are mentally simulated. Second, consequences are inferred from these simulations. Third, adaptive behavior and learning geared toward future planning and problem-solving occurs. The default network figures prominently in the explanation of (at least) the first of these processes (Figure 2).

As this example illustrates, grasping the relationships between different kinds of explanations can advance scientists’ understanding. In Figure 2, a computational account of mental simulation explains certain aspects of counterfactual reasoning, but mental simulation is then explained mechanistically: the default network consists of parts (e.g., ventral medial prefrontal cortex and posterior cingulate cortex) whose activities and interactions (anatomical connections) are organized so as to be responsible for various phenomena related to mental simulations. Quite plausibly, scientific understanding increases when the relationship between these two explanations is discovered.

**FIGURE 3:**
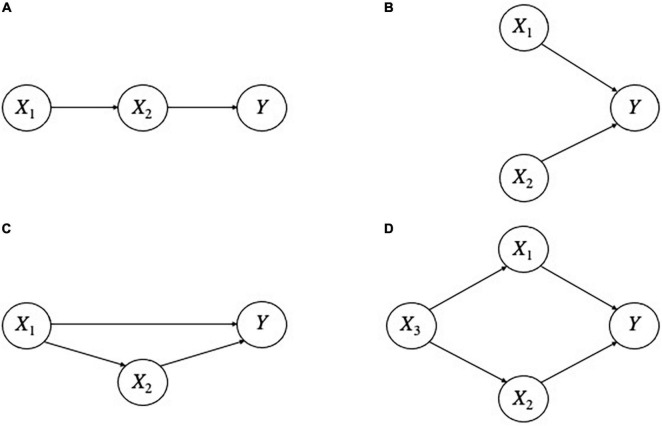
Different inter-explanatory relationships. Letters at the head of an arrow denote phenomena to be explained; those at the tail, factors that do the explaining. Thus, *X1* explains *X2* and *X2* explains *Y* in **(A)**; *X1* and *X2* independently explain *Y* in **(B)**. *X1* explains both *X2* and *Y*, and *X2* also explains *Y* in **(C)**; *X3* explains both *X1* and *X2*, which in turn each explain *Y* in **(D)**.

Importantly, this is but an instance of an indefinite number of other structures consisting of inter-explanatory relationships (see Figure 3 for examples). In all of these structures, we assume that for all *i, X*_*i*_ is a correct explanation of its respective explanandum. Intuitively, a person who could not distinguish these different explanatory structures would not understand *Y* as well as someone who did. For instance, a person who knew that *X*_1_ only explains *Y* through *X*_2_ in Figure 3A, or that *X*_1_ and *X*_2_ are independent of each other in Figure 3B, or that *X*_3_ is a common explanation or “deep determinant” of both *X*_1_ and *X*_2_ in Figure 3D, etc. seems to have a better understanding than a person who did not grasp these relationships. Undoubtedly, explanations can stand in other relationships that figure in the nexus.

Thus, the Nexus Principle provides useful guidelines for how different kinds of explanations should be integrated. Moreover, we have already seen that different kinds of explanations can stand in fruitful inter-explanatory relationships, and that these relationships enhance our understanding. In some cases, we may find that one and the same phenomenon is explained both mechanistically and non-mechanistically, but one of these explanations will be better than another. As noted above, “better than” and “worse than” are also inter-explanatory relationships. So, the Nexus Principle implies that knowing the relative strengths and weaknesses of different explanations enhances understanding.

The Scientific Knowledge Principle also plays a role in UBI. Suppose that *X*_1_ and *X*_2_ are competing explanations of *Y*. SEEing would largely be achieved when, through empirical testing, *X*_1_ was found to explain significantly more of *Y*’s variance than *X*_2_. This gives scientists grounds for thinking *X*_1_ better explains *Y* than *X*_2_ and thereby bolsters their understanding of *Y*. Importantly, SEEing is also how scientists discover other inter-explanatory relationships. An example is the aforementioned study that identified the inter-explanatory relationships between episodic memory, semantic memory, the default network, and mental simulation (Parikh et al., 2018).

### Mechanism-Based Integration

Aside from UBI, several other philosophical accounts of explanatory integration in the cognitive sciences are available (Kaplan, 2017; Miłkowski and Hohol, 2020). We provide some preliminary comparisons with the most prominent of these accounts, which we call *mechanism-based integration* (MBI). According to *strong* MBI, all models in the cognitive sciences are explanatory only insofar as they provide information about mechanistic explanations. In response, several critics of MBI—whom we call *pluralists—*have provided examples of putatively non-mechanistic explanations (see Table 2). When presented with putatively non-mechanistic explanations, e.g., of the computational, topological, and dynamical varieties, mechanists (i.e., MBI’s proponents) have two strategies available. First, the negative strategy argues that closer scrutiny of the relevant sciences reveals the putatively non-mechanistic explanation to be no explanation at all (Kaplan, 2011; Kaplan and Craver, 2011). The assimilation strategy argues that closer analysis of the relevant sciences reveals the putatively non-mechanistic explanation to be a mechanistic explanation, often of an elliptical nature (Piccinini, 2006, 2015; Piccinini and Craver, 2011; Zednik, 2011; Miłkowski, 2013; Povich, 2015; Hochstein, 2016). Mechanists inclined toward strong MBI frequently use the negative and assimilation strategies in a divide-and-conquer-like manner: the negative strategy applies to some putatively non-mechanistic explanations and the assimilation strategy applies to the rest. However, more prevalent is a *modest* form of MBI that simply applies these strategies to *some* putatively non-mechanistic explanations.

**TABLE 2:** Putatively non-mechanistic explanations discussed by philosophers.

Explanans	Explanandum	Scientific example	Philosophical work discussing example
**Computational explanations**			
Difference of Gaussians	Stereoscopic vision	Rodieck, 1965; Marr, 1982	Shagrir, 2010; Kaplan, 2011; Kaplan and Craver, 2011*; Bechtel and Shagrir, 2015; Rusanen and Lappi, 2016; Egan, 2017; Shapiro, 2019
Exhaustive search	Recall (memory)	Sternberg, 1969	Shapiro, 2017, 2019
Gain field encoding	Hand–eye coordination	Zipser and Andersen, 1988; Pouget and Sejnowski, 1997; Pouget et al., 2002; Shadmehr and Wise, 2005	Shagrir, 2006*; Kaplan, 2011*; Serban, 2015; Rusanen and Lappi, 2016; Egan, 2017
Geon composition	Object recognition	Hummel and Biederman, 1992	Weiskopf, 2011; Buckner, 2015*; Povich, 2015*
Hybrid computation	Efficiency of brain	Sarpeshkar, 1998	Chirimuuta, 2018
Inhibitory feedback	Normalization	Carandini and Heeger, 2012	Chirimuuta, 2014; Serban, 2015
Internal integration	Eye movement	Seung et al., 2000	Egan, 2017
Line attractor of choice axis, stimuli’s selection vector	Context-dependent decision making	Mante et al., 2013	Chirimuuta, 2018
Mapping non-coplanar points to unique rigid configuration	Three-dimensional visual structure of moving objects	Ullman, 1979	Shagrir and Bechtel, 2014*; Egan, 2017
Optimization of spatial and spectral information recovery (Gabor function)	V1 receptive fields	Daugman, 1985	Chirimuuta, 2014, 2018
Similarity of stimulus to stored exemplars	Categorization	Love et al., 2004; Kruschke, 2008	Weiskopf, 2011; Buckner, 2015*; Povich, 2015*
**Topological explanations**			
Closeness centrality	Speech and tonal processing	Mišić et al., 2018	Kostić, 2020
Mean connectivity	Ictogenicity	Helling et al., 2019	Kostić and Khalifa, 2021
Motif frequency	Functional connectivity	Adachi et al., 2011	Kostić and Khalifa, 2021, 2022 (see text footnote 4)
Navigation efficiency, diffusion efficiency	Efficiency of neuronal communication	Seguin et al., 2019	Kostić, 2020
Network communicability	Cognitive control	Gu et al., 2015	Kostić, 2020
Small-worldness	Information propagation	Watts and Strogatz, 1998	Kostić and Khalifa, 2022 (see text footnote 4)
**Dynamical explanations**			
Coupling of eye and bodily movements	Onset of motor control	Kelso et al., 1998; Shenoy et al., 2013	Chemero and Silberstein, 2008; Vernazzani, 2019*; Favela, 2020b
Coupling ratio	Bimanual coordination (relative phase)	Haken et al., 1985	Chemero, 2000, 2001; Kaplan and Craver, 2011*; Stepp et al., 2011; Zednik, 2011*; Lamb and Chemero, 2014; Golonka and Wilson, 2019*; Meyer, 2020
Strength of memory trace, salience of target, waiting time, stance	Infant reaching (A-not-B error)	Thelen et al., 2001	Zednik, 2011*; Gervais, 2015; Verdejo, 2015; Venturelli, 2016; van Eck, 2018*; Meyer, 2020; Povich, in press*
Potassium and sodium ion flows	Neural excitability	Hodgkin and Huxley, 1952; FitzHugh, 1961; Nagumo et al., 1962	Craver and Kaplan, 2011*; Kaplan and Bechtel, 2011*; Kaplan and Craver, 2011*; Ross, 2015; Hochstein, 2017*; Favela, 2020a,b

*The explanans (first column) is the factor that explains. The explanandum (second column) is the phenomenon to be explained. An asterisk indicates that the author takes the explanation to be mechanistic.*

Modest MBI diverges from pluralism on a case-by-case basis. Such cases consist of an explanation where the negative or assimilation strategy seems apt but stands in tension with other considerations that suggest the model is both explanatory and non-mechanistic. On this latter front, several pluralists argue that computational, topological, and dynamical explanations’ formal and mathematical properties are not merely abstract representations of mechanisms (Weiskopf, 2011; Serban, 2015; Rusanen and Lappi, 2016; Egan, 2017; Lange, 2017; Chirimuuta, 2018; Darrason, 2018; Huneman, 2018; van Rooij and Baggio, 2021). Others argue that these explanations cannot (Chemero, 2009; Silberstein and Chemero, 2013; Rathkopf, 2018) or need not (Shapiro, 2019) be decomposed into mechanistic components or that they cannot be intervened upon in the same way that mechanisms are intervened upon (Woodward, 2013; Meyer, 2020; Ross, 2020). Some argue that these putatively non-mechanistic explanations are non-mechanistic because they apply to several different kinds of systems that have markedly different mechanistic structures (Chirimuuta, 2014; Ross, 2015). Pluralist challenges specific to different kinds of explanations can also be found (e.g., Kostić, 2018; Kostić and Khalifa, 2022)^4^.

In what follows, we will show how UBI is deserving of further consideration because it suggests several plausible alternatives to the assimilation and negative strategies. As such, it contrasts with both strong and modest MBI. While we are partial to pluralism, our discussion here is only meant to point to different ways in which mechanists and pluralists can explore the issues that divide them. Future research would determine whether UBI outperforms MBI.

#### Assimilation Strategy

According to mechanists’ assimilation strategy, many putatively non-mechanistic explanations are in fact elliptical mechanistic explanations or “mechanism sketches” (Piccinini and Craver, 2011; Zednik, 2011; Miłkowski, 2013; Piccinini, 2015; Povich, 2015, in press). Thus, when deploying the assimilation strategy, mechanists take computational, topological, and dynamical models to fall short of a (complete) mechanistic explanation, but to nevertheless provide important information about such mechanistic explanations. Mechanists have proposed two ways that putatively non-mechanistic explanations can provide mechanistic information, and thereby serve as mechanism sketches. First, putatively non-mechanistic explanations can be *heuristics* for discovering mechanistic explanations. Second, putatively non-mechanistic explanations can *constrain* the space of acceptable mechanistic explanations.

An alternative interpretation is possible. The fact that non-mechanistic models assist in the identification of mechanistic explanations does not entail that the former is a species of the latter. Consequently, putatively non-mechanistic explanations can play these two roles with respect to mechanistic explanations without being mere mechanism sketches. In other words, “genuinely” *non-mechanistic* explanations can guide or constrain the discovery of *mechanistic* explanations. Earlier explanatory pluralists (McCauley, 1986, 1996) already anticipated precursors to this idea, but did not tie it explicitly as a response to mechanists’ assimilation strategy.

Moreover, this fits comfortably with our account of SEEing and hence with UBI. Heuristics of discovery are naturally seen as advancing SEEing’s first stage of considering plausible potential explanations. Similarly, since the goal of SEEing is to identify correct explanations and their relationships, it is a consequence of UBI that different kinds of explanations of the related phenomena constrain each other. For instance, suppose that we have two computational explanations of the same phenomenon, and that the key difference between them is that only the first of these is probable given the best mechanistic explanations of that phenomenon. Then that counts as a reason to treat the first computational explanation as better than the second. Hence, SEEing entails mechanistic explanations can constrain computational explanations.

More generally, UBI can capture the same key inter-explanatory relationships that mechanists prize without assimilating putatively non-mechanistic explanations to mechanistic explanation. Indeed, like many mechanists, UBI suggests that not only do putatively non-mechanistic explanations guide and constrain the discovery of mechanistic explanations, but that the converse is also true. (The next section provides an example of this.) Parity of reasoning entails that mechanistic explanations should thereby be relegated to mere “computational, topological, and dynamical sketches” in these cases, but mechanists must resist this conclusion on pain of contradiction. Since UBI captures these important inter-explanatory relationships without broaching the more controversial question of assimilation, it need not determine which models are mere sketches of adequate explanations. Future research would evaluate whether this is a virtue or a vice.

#### Negative Strategy

Mechanists’ assimilation strategy becomes more plausible than the UBI-inspired alternative if there are good grounds for thinking that the criteria that pluralists use to establish putatively non-mechanistic explanations as genuine explanations are insufficient. This is the crux of the mechanists’ negative strategy. As with the assimilation strategy, we suggest that UBI provides a suggestive foil to the negative strategy.

The negative strategy’s key move is to identify a set of non-explanatory models that pluralists’ criteria would wrongly label as explanatory. Two kinds of non-explanatory models—how-possibly and phenomenological models—exemplify this mechanist argument. How-possibly models describe factors that *could* but do not *actually* produce the phenomenon to be explained. For instance, most explanations begin as conjectures or untested hypotheses. Those that turn out to be false will be how-possibly explanations. Phenomenological models, which accurately describe or predict the target phenomenon without explaining it, provide a second basis for the negative strategy. Paradigmatically, phenomenological models correctly represent non-explanatory correlations between two or more variables. Mechanists claim that pluralist criteria of explanation will wrongly classify some how-possibly and some phenomenological models as correct explanations. By contrast, since models that accurately represent mechanisms are “how-actually models,” i.e., models that cite explanatory factors responsible for the phenomenon of interest, MBI appears well-positioned to distinguish correct explanations from how-possibly and phenomenological models.

However, UBI can distinguish correct explanations from how-possibly and phenomenological models. Moreover, it can do so in two distinct ways that do not appeal to mechanisms. First, it can do so on what we call *structural* grounds, i.e., by identifying non-mechanistic criteria of explanation that are sufficient for funding the distinction. It can also defuse the negative strategy on what we call *procedural* grounds, i.e., by showing that the procedures and methods that promote understanding also distinguish correct explanations from these non-explanatory models.

##### Structural Defenses

We suggest that the following provides a structural defense against the negative strategy:

If *X* correctly explains *Y*, then the following are true:

(1) *Accuracy Condition: X* is an accurate representation, and
(2) *Counterfactual Condition*: Had the objects, processes, etc. represented by *X* been different, then *Y* would have been different.

These are only necessary conditions for correct explanations. They are also sufficient for distinguishing correct explanations from how-possibly and phenomenological models but are likely insufficient for distinguishing correct explanations from every other kind of non-explanatory model. Identifying these other models is a useful avenue for future iterations of the negative strategy and responses thereto.

Situating this within UBI, these conditions are naturally seen as elaborating the Explanatory Floor, which claims that understanding a phenomenon requires possession of a correct explanation. Crucially, mechanists and pluralists alike widely accept these as requirements on correct explanations, though we discuss some exceptions below. Reasons for their widespread acceptance becomes clear with a simple example. Consider a case in which it is hypothesized that taking a certain medication (*X*) explains recovery from an illness (*Y*). If it were discovered that patients had not taken the medication, then this hypothesis would violate the accuracy condition. Intuitively, it would not be a correct explanation, but it would be a how-possibly model.

More generally, how-possibly models are correct explanations *modulo* satisfaction of the accuracy condition. Consequently, pluralists can easily preserve this distinction without appealing to mechanisms; accuracy is sufficient. Just as mechanisms can be either accurately or inaccurately represented, so too can computations, topological structures, and system dynamics be either accurately or inaccurately represented. Similarly, just as inaccurate mechanistic models can be how-possibly models but cannot be correct explanations, so too can inaccurate computational, topological, and dynamical models be how-possibly models but cannot be how-actually models.

Analogously, the counterfactual condition preserves the distinction between correct explanations and phenomenological models. Suppose that our hypothesis about recovery is confounded by the fact that patients’ recovery occurred 2 weeks after the first symptoms, and that this is the typical recovery time for anyone with the illness in question, regardless of whether they take medication. Barring extenuating circumstances, e.g., that the patients are immunocompromised, these facts would seem to cast doubt upon the claim that the medication made a difference to their recovery. In other words, they cast doubt on the following counterfactual: had a patient not taken the medication, then that patient would not have recovered when she did. Consequently, the hypothesis about the medication explaining recovery violates the counterfactual condition. Moreover, the hypothesis does not appear to be correct, but would nevertheless describe the patients’ situation, i.e., it would be a phenomenological model.

More generally, phenomenological models are correct explanations *modulo* satisfaction of the counterfactual condition. Just as a mechanistic model may accurately identify interacting parts of a system that correlate with but do not explain its behavior, a non-mechanistic model may accurately identify computational processes, topological structures, and dynamical properties of a system that correlate with but do not explain its behavior. In both cases, the counterfactual condition accounts for the models’ explanatory shortcomings; no appeal to mechanisms is needed.

##### Procedural Defenses

Admittedly, structural defenses against the negative strategy are not unique to UBI; other pluralists who are agnostic about UBI have invoked them in different ways. By contrast, our second *procedural* defense against the negative strategy is part and parcel to UBI. Procedural defenses show that the procedures that promote understanding also distinguish correct explanations from how-possibly and phenomenological models.

The Scientific Knowledge Principle characterizes the key procedures that simultaneously promote understanding and distinguish correct explanations from these non-explanatory models. Recall that SEEing consists of three stages: *considering* plausible potential explanations of a phenomenon, *comparing* them using the best available methods, and forming *commitments* to explanatory models based on these comparisons. This provides a procedural defense against the negative strategy. How-possibly and phenomenological models will only be acceptable in the first stage of SEEing: prior to their deficiencies being discovered, they frequently deserve *consideration* as possible explanations of a phenomenon. By contrast, correct explanations must “survive” the remaining stages of SEEing: they must pass certain empirical tests at the stage of comparison such that they are acceptable at the stage of commitment. Indeed, it is often through SEEing that scientists come to distinguish correct explanations from how-possibly and phenomenological models.

Crucially, consideration is most effective when it does not prejudge what makes something genuinely explanatory. This minimizes the possibility of missing out on a fruitful hypothesis. Consequently, both mechanistic and non-mechanistic explanations should be included at this initial stage of SEEing. However, our procedural defense supports pluralism only if some computational, topological, or dynamical explanations are acceptable in light of rigorous explanatory comparisons. As we see it, this is a strength of our procedural defense, for it uses the empirical resources of our best science to adjudicate debates between mechanists and pluralists that often appear intractable from the philosophical armchair.

Nevertheless, we can point to an important kind of explanatory comparison—which we call *control-and-contrast—*that deserves greater philosophical and scientific attention when considering explanatory integration in the cognitive sciences. Control-and-contrast proceeds as follows. Let *X*_1_ and *X*_2_ be two potential explanations of *Y* under consideration. Next, run two controlled experiments: one in which the explanatory factors in *X*_1_ are absent but those in *X*_2_ are present and the second in which the explanatory factors in *X*_1_ are present but those in *X*_2_ are absent. If *Y* is only present in the first experiment, then the pair of experiments suggests that *X*_2_ is a better explanation of *Y* than *X*_1_. Conversely, if *Y* is only present in the second experiment, the pair of experiments suggests that *X*_1_ is a better explanation of *Y* than *X*_2_. If *Y* is present in both experiments, the experiments are inconclusive. If *Y* is absent in both experiments, then the experiments suggest that the combination of *X*_1_ and *X*_2_ better explains *Y* than either *X*_1_ or *X*_2_ does in isolation. Since we suggest that both mechanistic and non-mechanistic explanations should be considered and thereby play the roles of *X*_1_ and *X*_2_, we also suggest that which of these different kinds of explanations is correct for a given phenomenon *Y* should frequently be determined by control-and-contrast.

In some cases, scientists are only interested in controlling-and-contrasting explanations of the same kind. However, even in these cases, the controls are often best described in terms of other kinds of explanation. For instance, as discussed above, the default mode network mechanistically explains mental simulations involved in episodic memory. By contrast, when mental simulations involve semantic memory, inferior temporal and lateral occipital regions play a more pronounced role (Parikh et al., 2018). Both episodic and semantic memory are functional or computational concepts that can figure as controls in different experiments designed to discover which of these mechanisms explains a particular kind of mental simulation. Less common is controlling-and-contrasting explanations of different kinds. Perhaps this is a lacuna in current research. Alternatively, it may turn out that different kinds of explanation rarely compete and are more amenable to integration in the ways outlined above.

The procedural defense complements the structural defense in two ways. First, not all pluralists accept the accuracy condition. Their motivations for this are twofold. First, given that science is a fallible enterprise, our best explanations today are likely to be refuted. Second, many explanations invoke idealizations, i.e., known inaccuracies that nevertheless enhance understanding. The procedural defense does not require the accuracy condition but can still preserve the distinction between correct explanations and non-explanatory models. Instead, the procedural defense only requires that correct explanations be acceptable on the basis of the best available scientific methods and evidence.

Second, tests such as control-and-contrast regiment the subjunctive conditionals that characterize the counterfactual condition. In evaluating counterfactuals, it is notoriously difficult to identify what must be held constant, what can freely vary without altering the truth-value of the conditional, and what must vary in order to determine the truth-value of the conditional. Our account of explanatory evaluation points to important constraints on this process. Suppose that we are considering two potential explanations *X*_*i*_ and *X*_*j*_ of some phenomenon *Y*. To compare these models, we will be especially interested in counterfactuals such as, “Had the value of *X*_*i*_ been different (but the value of *X*_*j*_ had remained the same), then the value of *Y* would have been different,” and also, “Had the value of *X*_*i*_ been different (but the value of *X*_*j*_ had remained the same), then the value of *Y* would have been the same.” These are precisely the kinds of counterfactuals that will be empirically supported or refuted by control-and-contrast.

## Conclusion

Fruitful connections between the philosophy and science of understanding can be forged. In a naturalized epistemology of understanding, philosophical claims about various forms of explanatory and counterfactual reasoning are empirically constrained by scientific tests and explanations. By contrast, in UBI, the philosophy of understanding contributes to the science of understanding by providing broad methodological prescriptions as to how diverse explanations can be woven together. Specifically, UBI includes identification of inter-explanatory relationships, consideration of different kinds of explanations, and evaluation of these explanations using methods such as control-and-contrast. As our suggestions have been of a preliminary character, we hope that future collaborations between philosophers and scientists will advance our understanding of understanding.

## Data Availability Statement

The original contributions presented in the study are included in the article/supplementary material, further inquiries can be directed to the corresponding author.

## Author Contributions

All authors contributed to conception and design of the study. Each author wrote at least one section of the manuscript. All authors contributed to manuscript revision, read, and approved the submitted version.

## Conflict of Interest

The authors declare that the research was conducted in the absence of any commercial or financial relationships that could be construed as a potential conflict of interest.

## Publisher’s Note

All claims expressed in this article are solely those of the authors and do not necessarily represent those of their affiliated organizations, or those of the publisher, the editors and the reviewers. Any product that may be evaluated in this article, or claim that may be made by its manufacturer, is not guaranteed or endorsed by the publisher.

## Footnotes

1 Petersen, S. (2022). *Explanation as Compression*.

2 Though see Osman (2004), Keren and Schul (2009), and Stephens et al. (2018) for examples of criticisms.

3 See Craver (2014) for an overview of the latter issue.

4 Kostić, D., and Khalifa, K. (2022). *Decoupling Topological Explanation from Mechanisms*.
